# Editorial: Sheep and Goat Gene Exploration

**DOI:** 10.3389/fgene.2022.802709

**Published:** 2022-03-31

**Authors:** Shaobin Li, Xin Wang, Rui Su

**Affiliations:** ^1^ Gansu Key Laboratory of Herbivorous Animal Biotechnology, Faculty of Animal Science and Technology, Lanzhou, China; ^2^ College of Animal Science and Technology, Northwest A & F University, Xianyang, China; ^3^ College of Animal Science, Inner Mongolia Agricultural University, Hohhot, China

**Keywords:** functional gene, phenotype, economic traits, goat, sheep

Domestic sheep (*Ovis aries*) and goats (*Capra hircus*) are valuable farm animals that provide meat, milk, and textile-fiber for people’s daily lives. Most of these traits are quantitative and controlled by multiple genes and environments. Elucidating the genetic and epigenetic mechanisms of these economic traits is critical to understanding how a trait comes into being. However, it is difficult and time-consuming work. The development of biotechnology and sequencing technology has accelerated the process of finding functional genes, which is beneficial to improving the economic traits of sheep and goat production.

Meat is the main product of sheep and goats, which is mainly affected by carcass traits (including fat), and some functional genes have been identified previously. *Myostatin* and *Callipyge* (*CLPG*) are two typical genes that can directly affect sheep carcass weight by changing hindquarters phenotype. The regulation of the expression of *MyHC* isoforms can change the myofiber types and thus change the meat quality in sheep (Bao et al.). The content of intramuscular fat (IMF) has a direct effect on the quality and flavor of the meat. A deficiency of Acetyl-CoA acyltransferase 1 (*ACAA1*) leads to an increases in the triglyceride content and lipid accumulation and promote differentiation of sheep preadipocytes, while with the overexpression of this gene a reverse phenomenon appears with a decrease of triglyceride content and lipid accumulation and inhibition of adipogenesis (Wang et al.). *APOA2*, *GALK1*, *ADIPOQ,* and *NDUFS4* may be involved in the deposition of fat in the tail of Chinese indigenous sheep (Zhu et al.). Some microRNAs (miRNAs) involved in adipocyte differentiation may be used to improve sheep meat quality and IMF content as new biomarkers (Xiao et al.), and miRNA-mRNA networking can co-regulate goat polyunsaturated fatty acid (PUFA) metabolism and synthesis (Xie et al.).

Wool and cashmere are some of the other important products of sheep and goat production. Some genes associated with the development of hair follicles or encoded fiber structural protein may have an effect on fiber traits. Besides, some miRNAs can also indirectly influence wool traits through regulating these genes. Fibroblast growth factors (FGF) and Wnt and some other signaling pathways have an important function in regulating the development of hair follicles (Harshuk-Shabso et al., 2020; Houschyar et al., 2020). *WNT2* could promote the growth and development of sheeps’ skin and hair follicles (Tian et al.). Further, some *FGF5* variations are associated with wool length, greasy wool weight, and mean fiber diameter in Fine wool sheep (Zhao et al.). Sulfur is a special component in animal fibers which mainly exists as organic sulfur-containing amino acids (SAAs), and has an important function on wool fiber quality. Melatonin may regulate sulfur metabolism by regulating genes related to the skin cell cycle and energy metabolism (Chai et al.), and it regulates cashmere growth via up-regulating*β-catenin* and *Wnt10b* expression (Liu et al.). circRNA may regulate the growth and development of hair follicles by the NF-kappa B signaling pathway and Notch signaling pathway in cashmere goats (Shang et al.).

Litter size is an important trait for muti-lamb sheep and goat breeds. Fecundity booroola (*FecB*) is a major gene on sheep prolificacy (Hua and Yang, 2009). A *FecB* variant shows moderate ovulation and litter size, and a shorter estrous cycle which can be highly recommended in sheep crossbreeding systems for commercial mutton production (Wang et al.). A 13-bp indel mutation in the 3′ UTR of A-kinase anchoring protein 12 gene (*AKAP12*) is significantly associated with litter size in Shanbei white cashmere goats of China (Kang et al.). miRNAs are related to the regulation of around 1/3 of all genes in mammals and are wildly involved in all kinds of biological and physiological processes including reproduction (Reza et al., 2019). chi-miR-324-3p inhibits the proliferation of goat granulosa cells by targeting *DENND1A* (Liu et al.). Some lncRNAs play a role in regulating cell division during ovarian development in goat (Li et al.) and may participate in the regulation of seasonal reproduction in sheep (Xia et al.).

In brief, this Research Topic highlights the diversity of functional genes and miRNAs related to sheep and goat economic traits, and their biological functions would be unraveled gradually. Furthermore, the establishment of some databases, eg iSheep (Wang et al.), is undoubtedly the icing on the cake.
